# Selective FRET nano probe based on carbon dots and naphthalimide–isatin for the ratiometric detection of peroxynitrite in drug-induced liver injury†

**DOI:** 10.1039/d3sc05010f

**Published:** 2023-12-14

**Authors:** Yueci Wu, Lu-Lu Sun, Hai-Hao Han, Xiao-Peng He, Weiguo Cao, Tony D. James

**Affiliations:** a Department of Chemistry, University of Bath Bath BA2 7AY UK t.d.james@bath.ac.uk; b Department of Chemistry, Shanghai University Shanghai 200444 P. R. China wgcao@shu.edu.cn; c Shandong Laboratory of Yantai Drug Discovery, Bohai Rim Advanced Research Institute for Drug Discovery Yantai Shandong 264117 P. R. China hanhaihao@simm.ac.cn; d Molecular Imaging Center, Shanghai Institute of Materia Medica, Chinese Academy of Sciences Shanghai 201203 P. R. China; e Key Laboratory for Advanced Materials, Joint International Research Laboratory of Precision Chemistry and Molecular Engineering, Feringa Nobel Prize Scientist Joint Research Center, Frontiers Center for Materiobiology and Dynamic Chemistry, School of Chemistry and Molecular Engineering, East China University of Science and Technology 130 Meilong Rd Shanghai 200237 P. R. China xphe@ecust.edu.cn; f The International Cooperation Laboratory on Signal Transduction, National Center for Liver Cancer, Eastern Hepatobiliary Surgery Hospital Shanghai 200438 P. R. China; g School of Chemistry and Chemical Engineering, Henan Normal University Xinxiang 453007 P. R. China

## Abstract

Drug-induced liver injury (DILI) is the most common cause for acute liver failure in the USA and Europe. However, most of DILI cases can recover or be prevented if treatment by the offending drug is discontinued. Recent research indicates that peroxynitrite (ONOO^−^) can be a potential indicator to diagnose DILI at an early stage. Therefore, the establishment of an assay to detect and track ONOO^−^ in DILI cases is urgently needed. Here, a FRET-based ratiometric nano fluorescent probe CD–N-I was developed to detect ONOO^−^ with high selectivity and excellent sensitivity. This probe consists of carbon dots and a naphthalimide–isatin peroxynitrite sensing system assembled based on electrostatic interactions. Using CD–N-I we were able to detect exogenous ONOO^−^ in live cells and endogenous ONOO^−^ in APAP-induced liver injury of HepG2 cells.

## Abstract 2

Ratiometric FRET-based probe CD–N-I was developed to detect exogenous ONOO^−^ in live cells and endogenous ONOO^−^ in APAP-induced liver injury of HepG2 cells.

## Graphical abstract

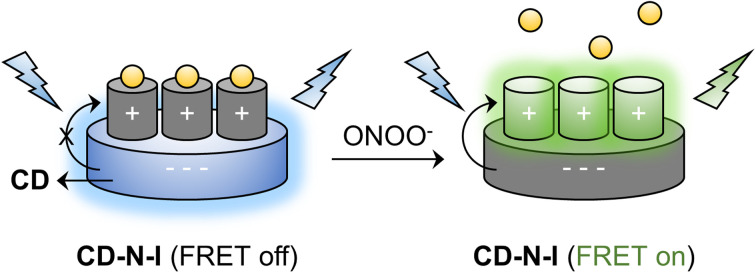

## Introduction

As a vital organ in the human body, the liver has multiple biological functions, including the immune response and vitamin storage.^1^ One of its key roles is to remove drugs from the body. This makes the liver a main target for drug-induced damage.^2^ Adverse drug reactions and overdoses are the main causes of drug-induced liver injury (DILI).^3,4^ Acetaminophen (APAP), as a typical analgesic and antipyretic drug, has been widely studied since a large number of DILI cases are related to the use of APAP.^5^ Recent studies have found that peroxynitrite (ONOO^−^) could be formed in vascular lumen during early stages of treatment or due to an overdose of APAP.^4^ As an early event in DILI, reactive metabolites of APAP can bind to proteins in hepatocytes, leading to mitochondrial oxidative stress.^4,6^ Then, due to mitochondrial dysfunction, the respiratory chain becomes inhibited, and the level of superoxide increases.^2,4,7^ When superoxide reacts with nitric oxide, ONOO^−^ is formed and promotes intracellular protein nitration.^4,8–10^ Meanwhile, with the continuous generation of intracellular ONOO^−^, parenchymal injury of hepatocytes is simultaneously aggravated.^4^ In addition, immune-mediated injury, as one critical mechanism of DILI, can not only lead to antibody-mediated cell death but also the release of reactive oxygen species (ROS) and cytokines, which results in hepatic injury.^2,11^ However, most DILI cases can recover or be prevented from developing into chronic liver disease and acute liver failure if treatment by the offending drug is discontinued.^2,12^ At the same time, ONOO^−^, as a ROS, can provide a potential indicator to diagnose drug-induced liver injury at an early stage. In general, up to 5 μM of ONOO^−^ exhibits a protective role towards the cardiovascular endothelium.^13^ However, when RAW 264.7 cells are exposed to 10–100 μM for 14 hours, cell apoptosis is observed.^14,15^ So, it is important to establish an assay to detect and track ONOO^−^ selectively, sensitively and rapidly, especially in DILI.

Currently, electrochemical sensors, nitrotyrosine detection and fluorescence monitoring are the most commonly used methods to detect ONOO^−^.^16,17^ Electrochemical sensors detect ONOO^−^ through electrocatalytic reduction.^16,17^ However, due to pH dependence of the peak potential and biocompatibility problems caused by implantation, the application of this approach is limited in complicated biological systems.^17–20^ The nitrotyrosine assay indirectly measures ONOO^−^ through the measurement of the concentration of nitrotyrosine formed by the nitration of tyrosine, determined using analytical approaches, such as HPLC, LC-MS or GC-MS.^21–24^ However, this method is not suitable for detecting ONOO^−^ in live cells. Compared to the former two approaches, fluorescence-based sensing exhibits the advantages of high sensitivity and selectivity, as well as the ability to directly monitor ONOO^−^ concentrations and localization in real time with live cells and *in vivo*.^25–27^ All these potential advantages make the detection method based on fluorescent probes a hot area of research for development.^28–37^ In general, fluorescent probes respond to target analytes based on specific interactions to generate changes in fluorescence intensity.^38–44^ However, detection methods based on emission intensities at a single wavelength are not reliable and accurate enough due to the interference from the environment and probe concentrations.^45–50^ Ratiometric fluorescent probes designed based on Föster resonance energy transfer (FRET) can avoid these kind of interference to a significant extent.^47,51^ Based on non-radiative diploe–dipole interaction, the energy of a fluorophore donor in an excited state can transfer to a fluorophore acceptor in the ground state.^52,53^ Due to the process of FRET, two fluorescent signals at different wavelengths can be measured at the same time. This method is more accurate because of the built-in correction and avoids interference caused by environmental and concentration changes.^45,53,54^

Carbon dots (CDs), are zero-dimensional nanomaterial with sizes typically less than 10 nm, consisting of sp^3^ hybridized carbon cores with partial sp^2^ hybridized carbon domains and shells rich with carboxyl, amino and hydroxyl groups.^55–59^ The photoluminescence of CDs is in part dependent on sp^2^ and sp^3^ carbon defects.^60–63^ Besides, CDs exhibit long absorption and emission wavelengths, large Stokes shift and excellent biocompatibility. In addition, due to their high fluorescence quantum yields, CDs can improve the sensitivity of the whole detection system. At the same time, their excellent water solubility and photostability can help inhibit the aggregation of fluorophores.^57,64,65^ Compared with traditional molecular fluorophores, the optical properties of CDs make them suitable as energy donors to construct FRET-based ratiometric fluorescent probes.^65,66^

**Scheme 1:**
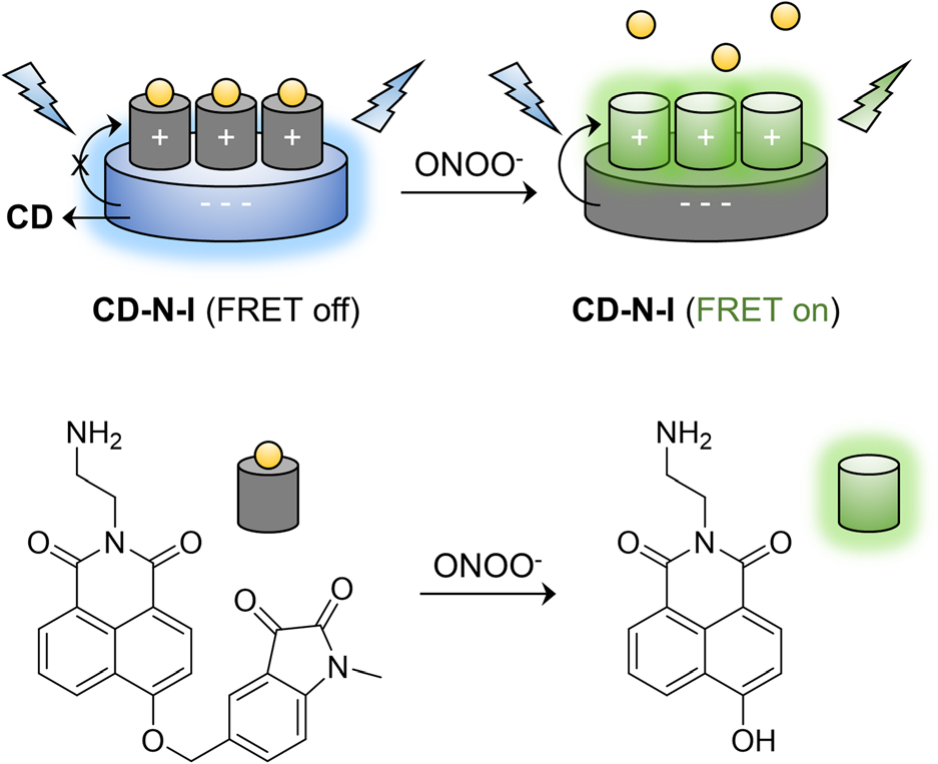
Schematic diagram of the mechanism of peroxynitrite detection using CD–N-I.

In our previous work, DSPE-PEG 2000 was used to encapsulate a naphthalimide–isatin peroxynitrite sensing system to construct an ICT-based turn-on fluorescent probe DSPE-PEG/HN-I to detect ONOO^−^ with high selectivity. However, the probe exhibited relatively poor imaging capability for endogenous ONOO^−^ in live cells.^67^ Consequently, inspired by the properties of CDs, we developed a FRET-based ratiometric nano probe CD–N-I to detect ONOO^−^ with high selectivity (Scheme 1). Therefore, with CD–N-I, CDs were used as energy donors and a naphthalimide fluorophore was used as an energy acceptor to construct an effective FRET platform based on electrostatic interactions. The addition of ONOO^−^ can trigger the activation of the isatin receptor and switch on the FRET process. This kind of ratiometric detection is more reliable due to reduced interference by environment factors. The supramolecular assembly of probes with CDs is a particularly simple approach by which to design FRET-type sensors as well as reducing the typical aggregation-caused quenching of naphthalimide based fluorescent probes. In addition, compared to using N-I alone, CD–N-I is more sensitive towards the detection of ONOO^−^. As such, CD–N-I could be used to image endogenous ONOO^−^ in APAP-induced live cells. These results confirm the potential of CD–N-I for the detection of ONOO^−^ in APAP-induced DILI.

## Results and discussion

### Preparation and characterization

**Fig. 1:**
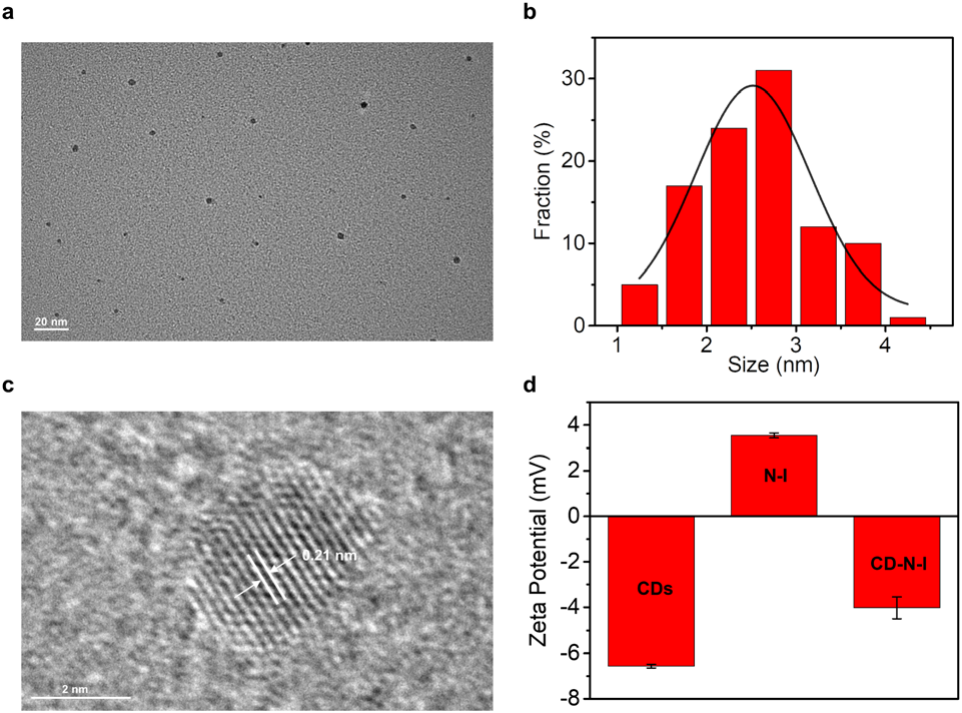
(a) TEM image of CDs. (b) The diameter distribution of CDs. (c) HR-TEM image of CDs. (d) Zeta potential of CDs (37.2 μg mL^−1^), N-I (147 μM) and CD–N-I (72.3 μg mL^−1^) in deionized water (containing 1% DMSO).

In order to obtain the nano probe CD–N-I for the detection of ONOO^−^, we independently synthesized CDs and the naphthalimide–isatin peroxynitrite sensing system N-I. Critic acid and ethylenediamine were heated at 180 °C for 1 hour to synthesize CDs based on the hydrothermal method.^65,68^ As shown by the TEM image (Fig. 1a and b), CDs were obtained as quasi-spherical structures and were well dispersed with diameters ranging from 1.3 nm to 4.5 nm. From Fig. 1c, the lattice spacing of CDs was about 0.21 nm. Based on the XPS spectrum of the CDs (Fig. S1a, ESI†), three main peaks at 532.2 eV, 399.1 eV and 284.7 eV correspond to O 1s, N 1s and C 1s respectively. From the high-resolution XPS spectrum of C 1s (Fig. S1b, ESI†), the peaks at 284.8 eV, 286.4 eV and 288.7 eV correspond to the C–C/C=C, C–O/C–N and C=O respectively. For the high-resolution XPS spectrum of O 1s (Fig. S1c, ESI†), the peaks at 532.4 eV and 532.5 eV correspond to the C=O and C–OH respectively. The high-resolution XPS spectrum of N 1s also shows two peaks at 398.2 eV and 399.4 eV corresponding to the C–N and N–H respectively (Fig. S1d, ESI†).^69,70^ The CDs were also characterized by FT-IR infrared spectroscopy. As shown in Fig. S2† (black line), the stretching vibration of O–H and N–H correspond to the absorption at 3000–3500 cm^−1^. Meanwhile, the stretching vibration C=O and C–N were observed at 1000–1750 cm^−1^. These results indicate that CDs are rich with carboxyl and amino groups.

Two absorptions at 245 nm and 350 nm in the UV-Vis spectrum of the CDs correspond to the transition of π–π* and n–π* (Fig. S3a, ESI†).^65,71^ As expected, CDs exhibit excitation-independent emission (Fig. S3b, ESI†). When the CDs were excited by wavelengths ranging from 350 nm to 420 nm, there was no obvious red shift of the emission spectra just decreasing fluorescence intensities. To date, the photoluminescent mechanism of CDs is still not fully understood. It is possible that conjugated π-domains and surface states tune the photoluminescence of the CDs.^72–75^ It has been suggested that the emission of the synthesized CDs follows Kasha Rules, which also explains their excitation-independent fluorescence behaviour.^76,77^

**Scheme 2:**
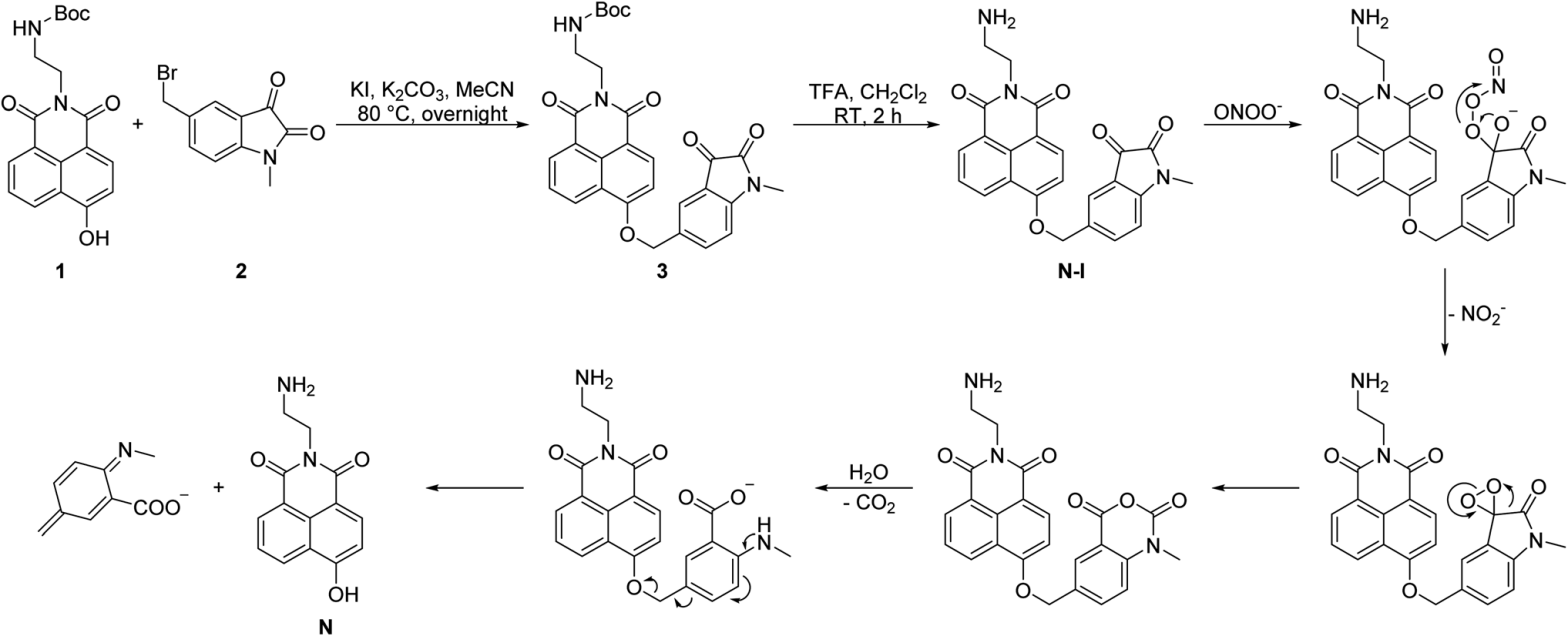
The synthetic route of N-I and its proposed reaction mechanism with ONOO^−^.

We readily obtained N-I (Scheme 2).^26,67,78–81^ The naphthalimide fluorophore with Boc protection 1 and the isatin group 2 were separately synthesized (Scheme S1, ESI†). Then, the two parts were linked by a nucleophilic reaction and compound 3 was obtained in a yield of 26%. TFA was then used to deprotect the Boc group and obtain N-I. All the compounds obtained were fully characterized using NMR, IR and HRMS.

A solution of CD–N-I was prepared by mixing two stock solutions of 2.48 mg per mL CDs and 9.78 mM N-I in PBS buffer (5.5 mM, pH = 7.4) with a ratio of 1 : 1 for 30 minutes.^65,82^ The zeta potential was used to confirm the assembly between the CDs and N-I (Fig. 1d). The zeta potential value of the CDs is −6.56 mV while the zeta potential value of N-I was 3.55 mV. After self-assembly, the zeta potential value of the nano probe CD–N-I was −4.02 mV. This value confirms the assembly of CDs and N-I was based on electrostatic interactions.^82–85^

### Spectral properties of CD–N-I

**Fig. 2:**
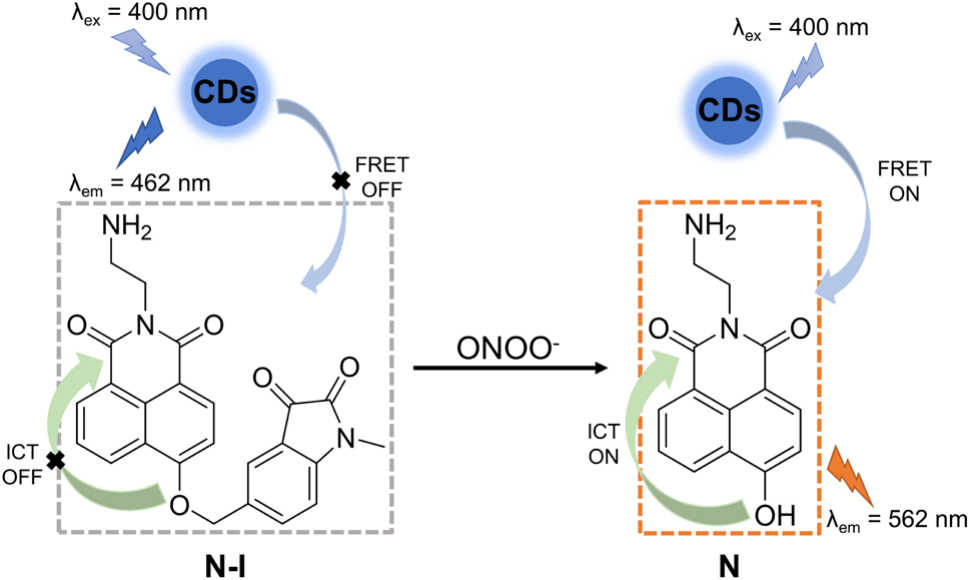
The proposed mechanism of CD–N-I with ONOO^−^.

**Fig. 3:**
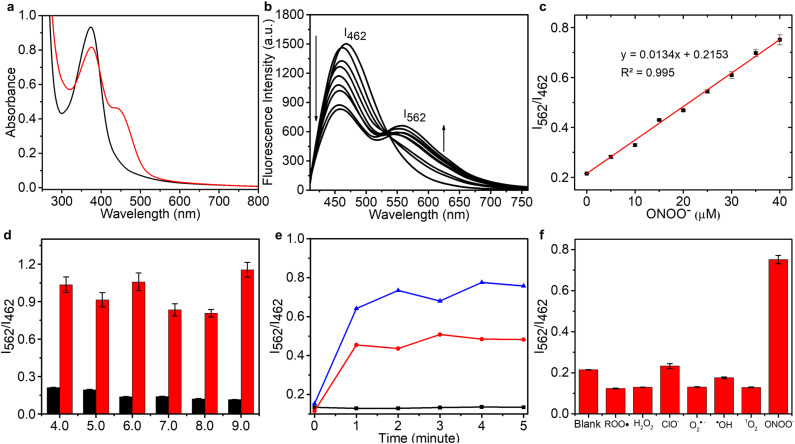
(a) UV-Vis spectra of 72.3 μg per mL CD–N-I without (black line) or with (red line) the addition of 40 μM ONOO^−^. (b) Emission spectra for CD–N-I (72.3 μg mL^−1^) in the presence of ONOO^−^ (0, 5, 10, 15, 20, 25, 30, 35, 40 μM). (c) Linear fluorescence ratio *I*_562_/*I*_462_ of CD–N-I towards ONOO^−^ (0–40 μM). (d) Effects pH on the fluorescence intensities of CD–N-I (72.3 μg mL^−1^) with (red bars) or without (black bars) the addition of ONOO^−^ (40 μM). (e) Time dependence of 72.3 μg per mL CD–N-I with the addition of ONOO^−^ at concentrations of 0 μM (black line), 20 μM (red line) and 40 μM (blue line). (f) Selectivity data for CD–N-I (72.3 μg mL^−1^) in the presence of ONOO^−^ (40 μM), ˙OH (500 μM), O_2_˙^−^ (500 μM), ^1^O_2_ (500 μM) after 5 minutes. H_2_O_2_ (1 mM), ROO˙ (500 μM) and ClO^−^ (500 μM) were measured after 30 minutes. The data was obtained in PBS buffer (5.5 mM, containing 1% DMSO), pH = 7.4 at 25 °C, *λ*_ex_ = 400 nm, *λ*_em_ = 462 nm and 562 nm.

In order to construct an efficient FRET platform between the CDs and N-I, the excitation wavelength of the CDs was selected according to the absorption wavelength of N-I. Based on UV-Vis and fluorescence spectra, when CDs were excited at 400 nm, there was a maximal overlap between the emission spectrum of the CDs and the absorption spectra of N-I (Fig. S4, ESI†). Therefore, 400 nm was selected as the most suitable excitation wavelength for CD–N-I. The fluorescence properties of CD–N-I were then evaluated in PSB buffer (5.5 mM, containing 1% DMSO) at pH = 7.4 and 25 °C. As shown in the UV-Vis spectrum (Fig. 3a), after the addition of ONOO^−^, a new absorption at 450 nm in addition to the original absorption at about 400 nm was observed. The fluorescence properties of CD–N-I were then investigated. Due to the isatin group, the ICT process of the naphthalimide fluorophore was inhibited so that the FRET process was in an off state (Fig. 2). So, there was only one emission at 462 nm when excited at 400 nm (Fig. 3b). As expected, upon the addition of ONOO^−^, cleavage of the isatin group turned on the ICT process of the naphthalimide fluorophore and switched on the FRET process from the CDs to N (Fig. 2).^67^ Due to the efficient energy transfer, a decreasing emission at 462 nm and an increasing emission at 562 nm with higher concentrations of ONOO^−^ was observed (Fig. 3b). For concentrations of ONOO^−^ ranging from 0 μM to 40 μM, the fluorescence intensity ratio *I*_562_/*I*_462_ increased linearly (Fig. 3c). The limit of detection of CD–N-I for ONOO^−^ detection was determined as 0.22 μM. Significantly, compared to using N-I alone, CD–N-I is more sensitive towards ONOO^−^, especially at low concentrations (Fig. S5, ESI†).

We then evaluated the pH sensitivity of CD–N-I by measuring the ratio of fluorescence intensities at *I*_562_/*I*_462_ in solutions with different pH values ranging from 4.0 to 9.0 (Fig. 3d). The results indicated that CD–N-I exhibited low sensitivity to pH changes, which confirms that it can be used in biological systems. Based on Fig. S6† and 3d, CD–N-I displayed good pH stability which was better than that observed for just CDs or N-I. As shown in Fig. 3e, about one minute was needed to complete the reaction. This result indicated that CD–N-I can respond rapidly to ONOO^−^. Results shown in Fig. S7† indicate good photostability of CD–N-I in the absence and presence of ONOO^−^ upon exposure to 365 nm light irradiation for 70 minutes. The thermal stability of the material was also evaluated (Fig. S8, ESI†). In the absence and presence of ONOO^−^, no obvious changes in the *I*_562_/*I*_462_ ratio of CD–N-I were observed over a temperature range from 25 °C to 45 °C, confirming its potential for use in cell imaging at 37 °C. Finally, the selectivity of CD–N-I was evaluated (Fig. 3f and S9, ESI†). Upon the addition of competing ROS and metal ions (ROO˙, H_2_O_2_, ClO^−^, O_2_˙^−^, ˙OH, ^1^O_2_, Fe^2+^, Fe^3+^, Cu^2+^, Zn^2+^, Co^2+^, Mn^2+^ and Se^4+^), minimal changes in the fluorescence intensity ratio *I*_562_/*I*_462_ were observed, which confirms the high selectivity of CD–N-I for ONOO^−^.

### Live cell imaging

**Fig. 4:**
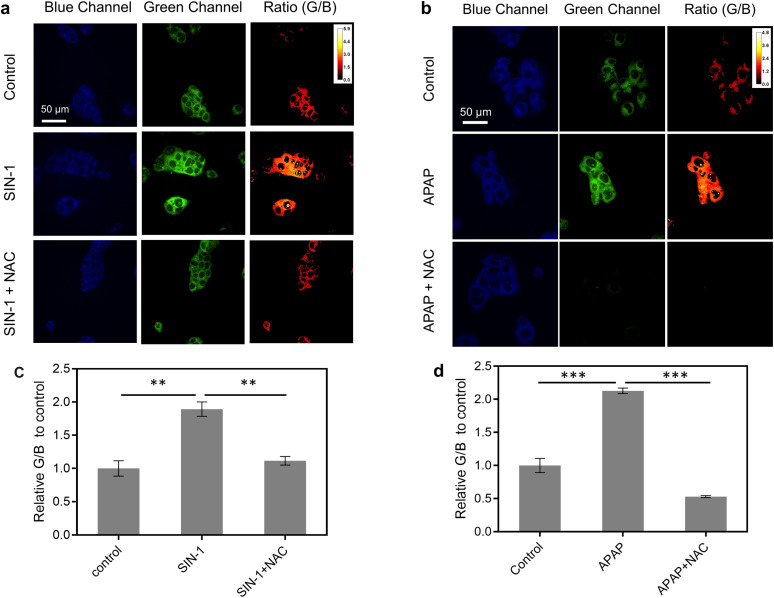
(a) Fluorescence imaging and (c) fluorescence quantification of HepG2 cells treated with the CD–N-I (72.3 μg mL^−1^, 2 h) in the absence and presence of SIN-1 (2 mM) and NAC (1 mM). Fluorescence data was collected using *λ*_ex_ = 405 nm, *λ*_em_ = 440–500 nm and 540–600 nm. **P < 0.01. Error bars represent S. D. (*n* = 3). (b) Fluorescence imaging and (d) fluorescence quantification of HepG2 cells treated with the CD–N-I (72.3 μg mL^−1^, 2 h) in the absence and presence of APAP (4 mM) and NAC (1 mM). Fluorescence data was collected using *λ*_ex_ = 405 nm, *λ*_em_ = 440–500 nm and 540–600 nm. ***P < 0.001. Error bars represent S. D. (*n* = 3).

Before imaging ONOO^−^ in live cells, the cell viability of CD–N-I was assessed in live HepG2 cells using a cell counting kit-8 (CCK-8) assay. After 72.3 μg per mL CD–N-I was incubated with the cells for 6 hours or 24 hours, minimal cell death was observed, which confirms the low cytotoxicity of CD–N-I (Fig. S10, ESI†). Then, CD–N-I was evaluated for imaging exogenous and endogenous ONOO^−^ respectively in HepG2 cells. Cells were pre-treated with 3-morpholinosydnonimine hydrochloride (SIN-1, a known ONOO^−^ donor) or *N*-acetylcysteine (NAC, an ONOO^−^ scavenger).^86,87^ We observed that when cells were pre-treated with SIN-1, the fluorescence intensity ratio (*I*_G_/*I*_B_) of the CD–N-I in HepG2 exhibited a significant concentration-dependent enhancement with respect to the control group (Fig. 4a and c and S11, ESI†). However, upon pre-treatment of cells with NAC, the increase in fluorescence intensity ratio (*I*_G_/*I*_B_) of CD–N-I that was induced by SIN-1 was attenuated (Fig. 4a and c).

Given the high expression of ONOO^−^ in cells induced by certain hepatotoxic drugs, we then investigated the applicability of using CD–N-I for the real-time monitoring of DILI. We used acetaminophen (APAP), a painkiller and antipyretic, to induce hepatocytes (HepG2 cells) a classic cell model for DILI research.^88^ Strong blue fluorescence and weak green fluorescence were observed in APAP-free HepG2 cells (Fig. 4b and d and S12, ESI†). However, pre-treatment of cells with APAP (4 mM) for 24 h and 48 h led to a significant time-dependent increase in the fluorescence ratio (G/B) of CD–N-I (Fig. S12, ESI†). To confirm that the fluorescence ratio (*I*_G_/*I*_B_) was triggered by the excessive production of ONOO^−^, HepG2 cells were preincubated with NAC, an agent that mitigates APAP-induced hepatotoxicity. The results aligned with our expectations, as there was a marked decrease in fluorescence intensity ratio (*I*_G_/*I*_B_) due to the reduced ONOO^−^ production (Fig. 4b and d). These findings demonstrate that CD–N-I can not only monitor and visualize the process of liver injury but could also be used to assess the efficacy of hepatoprotective drugs in hepatocytes.

## Conclusions

In brief, we have developed a self-assembled FRET-based ratiometric nano probe CD–N-I formed by electrostatic interactions between CDs and a naphthalimide–isatin peroxynitrite sensing system. On reaction with ONOO^−^, the isatin is cleaved which switches on the FRET process from the CDs to the naphthalimide fluorophore. Based on the sensing performance in solution and live cells, CD–N-I exhibits high selectivity and excellent sensitivity towards ONOO^−^ over other ROS. At the same time, due to its low cytotoxicity, excellent water solubility and photostability, CD–N-I was successfully used to image exogenous and endogenous ONOO^−^ in live cells. In particular, the imaging ability of CD–N-I in APAP-induced cells indicates that the probe has great potential for applications in evaluating DILI.

## Data availability

The authors confirm that the data supporting the findings of this study are available within the article ESI.†

## Author contributions

Yueci Wu: conceptualization, data curation, formal analysis, investigation, methodology, validation, visualization, writing – original draft, writing – review & editing. Lu-Lu Sun: data curation, formal analysis, investigation, methodology, validation, visualization, writing – original draft. Hai-Hao Han: funding acquisition, project administration, resources, supervision, writing – original draft, writing – review & editing. Xiao-Peng He: funding acquisition, project administration, resources, supervision, writing – original draft, writing – review & editing. Weiguo Cao: funding acquisition, project administration, resources, supervision, writing – original draft, writing – review & editing. Tony D. James: conceptualization, funding acquisition, methodology, project administration, supervision, visualization, writing – original draft, writing – review & editing.

## Conflicts of interest

There are no conflicts to declare.

## Supplementary Material

SC-015-D3SC05010F-s001
