# Supplementary material for: Selective FRET nano probe based on carbon dots and naphthalimide–isatin for the ratiometric detection of peroxynitrite in drug-induced liver injury
Source: Chem Sci. 2023 Dec 14;15(2):757–64. doi: 10.1039/d3sc05010f (PMC10762965; doi:10.1039/d3sc05010f)
Supplement: SC-015-D3SC05010F-s001 [file SC-015-D3SC05010F-s001.pdf]

Electronic Supplementary Material (ESI) for Chemical Science.
This journal is © The Royal Society of Chemistry 2023

Supplementary Information

Selective FRET nano probe based on carbon dots and naphthalimide-isatin for the ratiometric detection of peroxynitrite in drug-induced liver injury

Yueci Wu [‡a], Lu-Lu Sun [‡c, d], Hai-Hao Han[*c,d], Xiao-Peng He[*e,f], Weiguo Cao[*b] and Tony D. James[*a,g].

a. Department of Chemistry, University of Bath, Bath, BA2 7AY, UK
b. Department of Chemistry, Shanghai University, Shanghai 200444, China
c. Shandong Laboratory of Yantai Drug Discovery, Bohai Rim Advanced Research Institute for Drug Discovery, Yantai, Shandong 264117, P. R. China
d. Molecular Imaging Center, Shanghai Institute of Materia Medica, Chinese Academy of Sciences, Shanghai 201203, P. R. China
e. Key Laboratory for Advanced Materials and Joint International Research Laboratory of Precision Chemistry and Molecular Engineering, Feringa Nobel Prize Scientist Joint Research Center, Frontiers Center for Materiobiology and Dynamic Chemistry, School of Chemistry and Molecular Engineering, East China University of Science and Technology, 130 Meilong Rd., Shanghai 200237, P. R. China
f. The International Cooperation Laboratory on Signal Transduction, National Center for Liver Cancer, Eastern Hepatobiliary Surgery Hospital, Shanghai 200438, P. R. China
g. School of Chemistry and Chemical Engineering, Henan Normal University, Xinxiang 453007, P. R. China

[‡] Equally contribution

Corresponding Author:
*Email: hanhaihao@simm.ac.cn;
*Email: xphe@ecust.edu.cn;
*Email: wgcao@shu.edu.cn;
*Email: t.d.james@bath.ac.uk;

Contents

1. Experimental Details....................S3

2. Characterization of CDs....................S8

3. Fluorescence Measurements....................S9

4. Cell Imaging....................S14

5. NMR....................S17

6. References....................S20

1. Experimental Details

1.1 General Experimental Information

Reagents and solvents were sourced from commercial suppliers and used directly as received. TLC was carried out on commercially available pre-coated aluminum-backed silica plates and compounds were visualized under UV light at 356 nm. Column chromatography was performed using 60 micron silica purchased from Sigma Aldrich. Melting points were recorded on a WRS-1 instrument and are uncorrected. ^{1}H NMR and ^{13}C NMR spectra were recorded either in deuterated chloroform or dimethylsulfoxide at ambient temperature on a Bruker Avance 500 (500 MHz), with proton decoupling for all ^{13}C NMR spectra. HR-MS (high resolution mass spectra) was obtained on Agilent 6230, Thermo Fisher Scientific LTO FT Ultra, Thermo DFS Simm 210954 and HP-5989 instruments. Cell viability data were recorded by EnVision multilabel plate reader (PerkinElmer, USA). The cell fluorescence imaging experiments were conducted on a confocal laser-scanning microscopy (Olympus FV3000, Tokyo, Japan).

1.2 Synthesis of **CD-N-I**

1.2.1 Synthesis of **N-I**

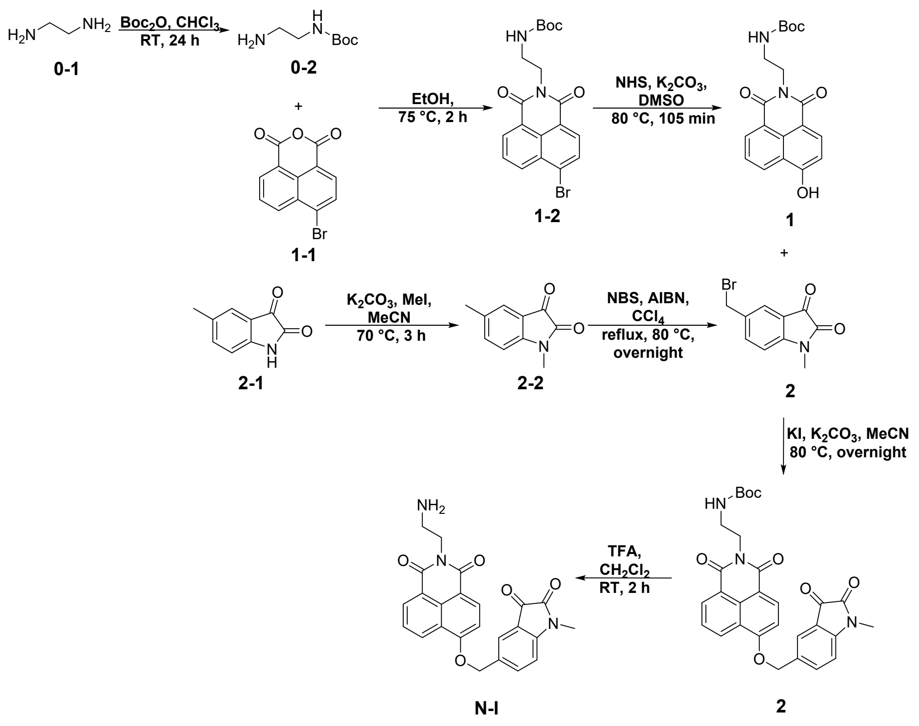

Scheme S1. The synthetic route of **N-I**.[1-6]

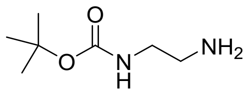

0-2

***Tert*-butyl (2-aminoethyl) carbamate**

Ethylenediamine (11 mL, 0.17 mol) was dissolved in chloroform (65 mL) to which di-*tert*-butyl dicarbonate (3.73 g, 0.017 mol) with the slow addition of chloroform (25 mL) at 0 °C. The mixture was stirred for 24 h at RT. Then, the mixture was poured into water. Brine (2 × 60 mL) was used to separate and wash the organic layer. Finally, after using $MgSO_4$ and evaporating in *vacuo*, compound **0-2** (1.8 g, 66 %) was obtained as a mixture of solid and clear oil.[7]

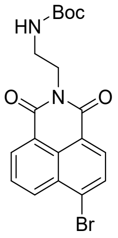

1-2

Tert-butyl (2-(6-bromo-1,3-dioxo-1H-benzo[de]isoquinolin-2(3H)-yl)ethyl)carbamate

4-Bromo-1,8-naphthalic anhydride (3.9 g, 13.9 mmol) and tert-butyl (2-aminoethyl) carbamate (2.2 g, 13.9 mmol) were dissolved in ethanol (60 mL). The mixture was stirred under reflux at 75 °C for 2 h. After the completion of the reaction, the solution was cooled to room temperature, and the solid filtered off. Finally, compound **1-2** (4.8 g, 96%) was obtained as an orange solid after being washed with cold ethanol and evaporated in vacuo.[8]

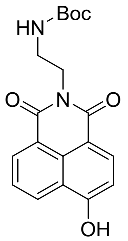

1

Tert-butyl (2-(6-hydroxy-1,3-dioxo-1H-benzo[de]isoquinolin-2(3H)-yl) ethyl) carbamate

Compound **1-2** (4.8 g, 13.5 mmol), N-hydroxysuccinimide (NHS) (1.7 g, 14.9 mmol) and K_2CO_3 (6.3 g, 44.8 mmol) were dissolved in DMSO (30 mL). The mixture was stirred at 80 °C for 105 minutes. After the completion of the reaction, the solution was cooled to room temperature, diluted with H_2O and acidified to pH = 1 with 1 M HCl. The precipitate was collected by vacuum filtration and washed with H_2O. Finally, compound **1** (3.0 g, 75%) was obtained as a yellow solid.

^{1}H NMR (500 MHz, DMSO): δ = 11.80 (s, 1 H), 8.50 (t, *J* = 9.3 Hz, 1 H), 8.43 (dd, *J* = 11.1 Hz, *J* = 6.9 Hz, 1 H), 8.32 (dd, *J* = 10.7 Hz, *J* = 8.3 Hz, 1 H), 7.73 (dd, *J* = 15.6 Hz, *J* = 8.0 Hz, 1 H), 7.13 (t, *J* = 8.1 Hz 1 H), 6.85 (t, *J* = 6.0 Hz,1 H), 4.09 (t, *J* = 6.0 Hz, 2 H), 3.22 (dd, *J* = 12.0 Hz, *J* = 6.0 Hz, 2 H), 1.2 (s, 9 H); ^{13} C NMR (126 MHz, DMSO): δ = 163.86, 163.17, 160.00, 155.69, 133.28, 130.88, 129.30, 128.61, 125.42, 122.30, 122.05, 112.90, 109.78, 77.40, 40.43, 39.46, 37.90, 28.10, 27.79 ppm; m.p.: 219-220 °C; IR (KBr): *υ* = 3473.74 (s, N-H), 3358.05 (s, O-H), 1700.41 (s, C=O), 1681.28 (s, C=O), 1647.75 (s, C=O), 1365.17 (s, C-O-C), 1240.68 (s, C-N); HRMS (ESI) calcd. for $C_{19}H_{20}N_2O_5$ $[M+H]^+$: 357.1372, found 357.1461.

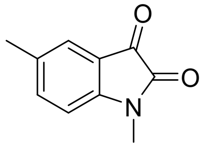

2-2

1,5-Dimethylindoline-2,3-dione

5-Methyl isatin (1.0 g, 6.2 mmol) and K_2CO_3 (1.72 g, 12.4 mmol) were dissolved in acetonitrile (15 mL), and then methyl iodide (1.32 g, 9.3 mmol) was added dropwise. The mixture was stirred at 70°C for 3 h. After the reaction was complete, the solution was extracted with ethyl acetate (30 mL ×3). The organic layer was dried using anhydrous Na_2SO_4 and concentrated under reduced pressure. Finally, compound **2-2** (0.97 g, 90%) was obtained as a red solid.[2]

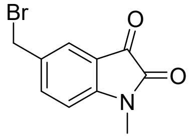

2-3

5-(Bromomethyl)-1-methylindoline-2,3-dione

Compound **2-2** (0.8 g, 4.6 mmol) and N-bromosuccinimide (NBS) (0.82 g, 4.6mmol) were dissolved in CCl_4 (30 mL), and the solution was heated at 80°C and reflux for 10 minutes, 2,2'-azobis(2-methylpropionitrile) (AIBN) (0.08 g, 0.5 mmol) was added and the mixture refluxed overnight. Finally, the solvent was removed under reduced pressure, and the mixture was purified by column chromatography on silica gel (petroleum ether/ ethyl acetate = 4:1). Compound **2-3** (0.12 g, 10%) was obtained as a red solid.[2]

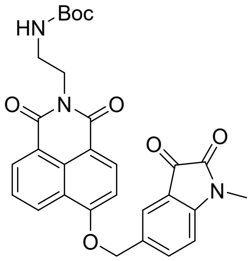

3

Tert-butyl (2-(6-((1-methyl-2,3-dioxoindolin-5-yl) methoxy)-1,3-dioxo-1H-benzo[de]isoquinolin-2(3H)-yl) ethyl) carbamate

Compound **1** (1.3 g, 4.3 mmol), compound **2** (1.1 g, 4.3 mmol), KI (56.6 mg, 0.3 mmol) and K_2CO_3 (0.6 g, 4.3 mmol) were dissolved in acetonitrile (60 mL). The solution was refluxed at 80 °C overnight. After the completion of the reaction, the solvent was evaporated in *vacuo*. Finally, compound **3** (0.6 g, 26%) was obtained as an orange solid by column chromatography on silica gel (dichloromethane / ethyl acetate = 4:1).

^{1}H NMR (500 MHz, DMSO): δ = 8.39 (t, *J* = 8.3 Hz, 2 H), 8.33 (d, *J* = 8.3 Hz 1 H), 7.86 (dd, *J* = 8.1 Hz, *J* = 1.8 Hz, 1 H), 7.72 (t, *J* = 7.7 Hz, 1 H), 7.69 (d, *J* = 1.8 Hz, 1 H), 7.33 (d, *J* = 8.4 Hz, 1 H), 7.16 (d, *J* = 8.1 Hz, 1 H), 6.85 (t, *J* = 6.1 Hz, 1 H), 5.37 (s,2 H), 4.07 (t, *J* = 5.9 Hz, 2 H), 3.22 (dd, *J* = 12.0 Hz, *J* = 6.0 Hz, 2 H), 3.13 (s, 3 H), 1.20 ppm (s, 9 H); ^{13}C NMR (126 MHz, DMSO): δ = 183.15, 163.66, 162.98, 158.76, 158.22, 158.22, 155.70, 151.24, 137.89, 132.72, 130.95, 130.79, 128.62, 127.93, 126.29, 123.92, 122.73, 117.47, 114.70, 110.68, 107.22, 77.40, 69.50, 39.26, 37.82, 28.07, 27.80, 26.09 ppm; m.p.: 224-226 °C; IR (KBr): υ = 1733 (s, C=O), 1654 (s, C=O), 1354 (s, C-O-C), 1262 (s, C-N); HRMS (ESI) calcd. for $C_{29}H_{27}N_3O_7$ $[M+H]^+$: 530.1849, found 530.1948.

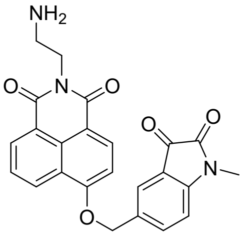

N-I

2-(2-Aminoethyl)-6-((1-methyl-2,3-dioxoindolin-5-yl) methoxy)-1H-benzo[de]isoquinoline-1,3(2H)-dione

Compound **3** (1.3 g, 2.4 mmol) was added in dichloromethane (10 mL) to which trifluoracetic acid (1.2 mL, 15.6 mmol) was added. The mixture was stirred at RT for 2 h. Then, the solvent was evaporated in *vacuo*. Finally, compound **N-I** (1.52 g, over quantitative yield) was acquired as an orange solid. Due to the addition of TFA, the final compound can probably be a mixture of **N-I** and its salt forming with TFA. This caused the final yield over quantitative.

^{1}H NMR (500 MHz, DMSO): δ = 8.50 (d, *J* = 8.5 Hz, 1 H), 8.47 (d, *J* = 7.3 Hz 1 H), 8.42 (d, *J* = 8.3 Hz 1 H), 7.92-7.89 (m, 3 H), 7.80 (t, *J* = 7.6 Hz, 1 H), 7.72 (d, *J* = 1.7 Hz, 1 H), 7.42 (d, *J* = 8.5 Hz, 1 H), 7.20 (d, *J* = 8.2 Hz, 1 H), 5.43 (s, 2 H), 4.29 (t, *J* = 5.9 Hz,2 H), 3.15 ppm (s, 5 H); ^{13} C NMR (126 MHz, DMSO): δ = 183.22, 164.20, 163.46, 159.13, 158.29, 151.32, 137.98, 133.07, 131.06, 130.95, 128.81, 128.37, 126.51, 123.98, 122.89, 122.12, 117.54, 114.66, 110.75, 107.44, 69.65, 37.68, 37.39, 26.13 ppm; m.p.: 159-160 °C; IR (KBr): *υ* = 3434.74 (s, N-H), 3166.99 (s, N-H), 1741.57 (s, C=O), 1721.50 (s, C=O), 1697.38 (s, C=O), 1660.65 (s, C=O), 1380.39 (s, C-O-C), 1272.27 (s, C-N); HRMS (ESI) calcd. for $C_{24}H_{19}N_{3}O_{5}$ $[M+H]^{+}$: 430.1325, found 430.1397.

1.2.2 Preparation of CDs

Based on published papers, hydrothermal synthesis was used to synthesize CDs.[9, 10] 2 g citric acid and 0.4 mL ethylenediamine were dissolved in 10 mL deionized water. Then, the solution was transferred to a 50 mL Teflonlined stainless autoclave and heated at 180 °C for 1 hour. After the reaction was completed, the brown powder was washed with water and ethyl alcohol and dried.

1.2.3 Preparation of **CD-N-I**

Based on published method, a solution of **CD-N-I** was prepared.[9, 11] A 9.78 mM stock solution of probe **N-I** was prepared in DMSO. At the same time, a 2.48 mg/mL stock of CDs was prepared in PBS buffer (5.5 mM, pH=7.4). Then, the two stock solutions were mixed with a ratio of 1:1 and incubated for 30 minutes to obtain the FREE-based ratiometric nano probe **CD-N-I**.

2. Characterization of CDs

2.1. General Experimental Information

TEM image was obtained on JEOL-JEM 2100 F Transmission Electron Microscope (Japan) by VAN-RESEARCH INTELLIGENCE TECHNOLOGY (ZHENGZHOU) CO. LTD. X-ray Photoelectron Spectra (XPS) was recorded on ESCALAB 250Xi (Thermofisher Scientific China., Ltd). Zeta potential was measured on a NanoBrook Omni (BrookHaven Instruments Corp., Holtsville, NY).

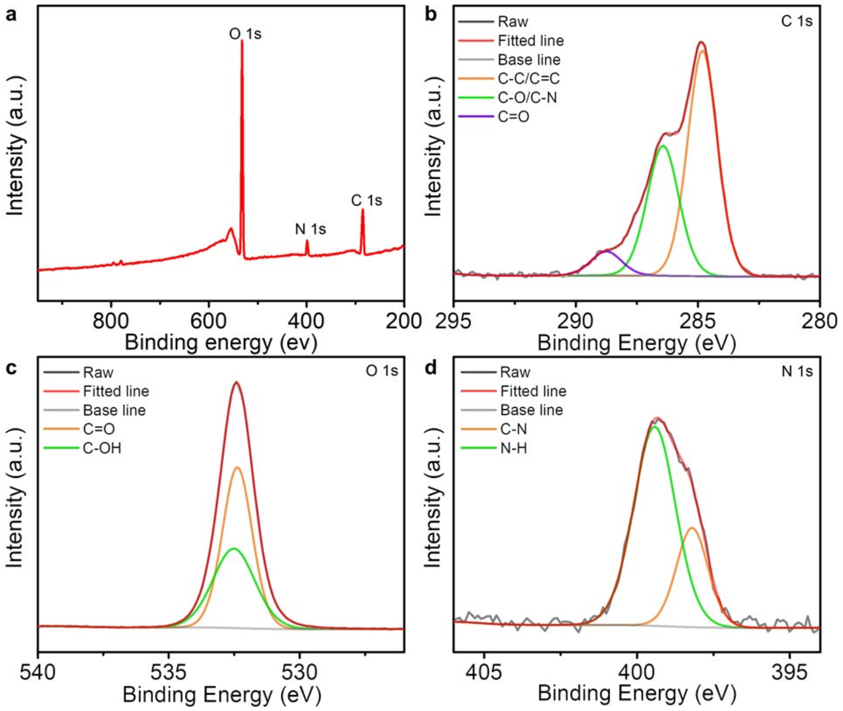

Figure S1. XPS spectra of CDs (**a**). High-solution XPS spectra of the C 1s (**b**), the O 1s (**c**) and the N 1s (**d**) of the CDs.

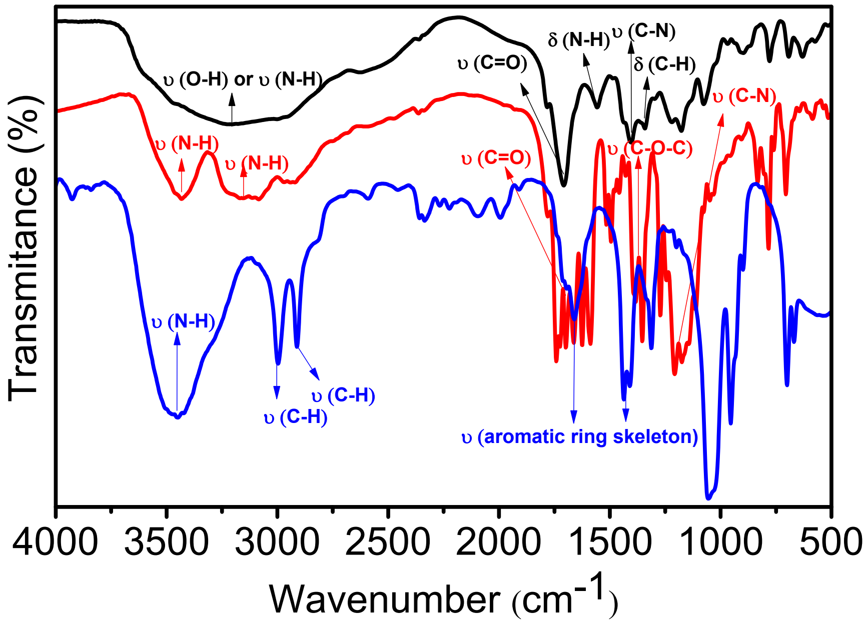

Figure S2. FTIR spectra of CDs (black line), **N-I** (red line) and **CD-N-I** (blue line).

3. Fluorescence Measurements

3.1. General Experimental Information

Fluorescence measurements were performed on a RF-5301PC Fluorescence Spectrophotometer (Shimadzu, Japan). All pH measurements taken during fluorescence/absorption experiments were recorded on a pH meter (Shanghai Leici instrument factory). UV-VIS measurements were performed on a UV-2501PC Visible Spectrophotometer (Shimadzu, Japan). Fluorescence quantum yields were measured on a Edinburgh Photoluminescence Spectrometer FLS 1000 (UK).

Phosphate buffered saline (PBS) was freshly prepared from 100% water with KCl (10 mM), KH_2PO_4 (2.752 mM) and Na_2HPO_4 (2.757 mM). The PBS buffer was adjusted to pH 7.4 with 1 M HCl (aq).

Hydrogen peroxide (H_2O_2) is commercially available whereby the concentration of H_2O_2 was determined through spectrophotometrical analysis with $\varepsilon = 43.6\ cm^{-1}\ M^{-1}$ at 240 nm.

Peroxynitrite ($ONOO^-$) stock solutions were freshly prepared each time prior to usage. A solution of 3 M NaOH was cooled to 0 °C to which simultaneously 0.7 M H_2O_2, 0.6 m $NaNO_2$ and 0.6 M

HCl were added. The $ONOO^-$ solution was analyzed spectrophotometrically whereby the concentration of $ONOO^-$ was estimated through $\varepsilon = 1670 \pm 50$ cm^{-1} M^{-1} at 302 nm in 0.1 M NaOH (aq.).

ROO• was generated from 2, 2'-azobis (2-amidinopropane) dihydrochloride. AAPH (2, 2‘azobis (2-amidinopropane) dihydrochloride, 0.1 M) was added into deionizer water, and then stirred at 37 °C for 30 min.

Superoxide was generated from KO_2. KO_2 (1.0 eq) and 18-crown-6 ether (2.5 eq) was dissolved in DMSO to afford a superoxide stock solution.

Hydroxyl radical was generated by the Fenton reaction. To prepare •OH solution, hydrogen peroxide (H_2O_2, 10 eq) was added to $Fe(ClO_4)_2$ in deionised water.

The concentration of ClO^- was determined from the absorption at 292 nm ($\mathcal{E}$ = 350 M^{-1} cm^{-1}).

3.2. Fluorescence and UV-VIS Spectra

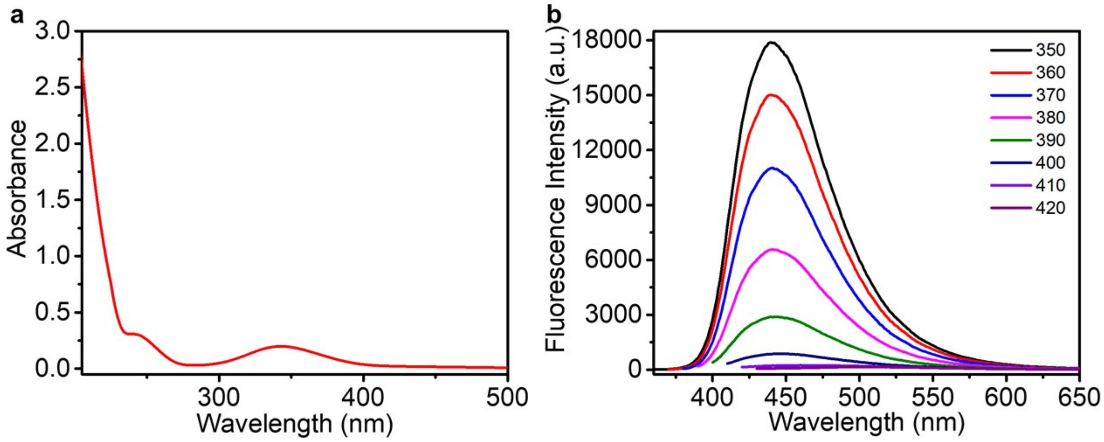

Figure S3. a. UV-VIS spectrum of CDs (37.2 μg/mL). **b.** Fluorescence emission spectra of CDs (37.2 μg/mL) at different excitation wavelengths from 350 nm to 420 nm. The data was obtained in PBS buffer (5.5 mM, containing 1% DMSO), pH=7.4 at 25°C.

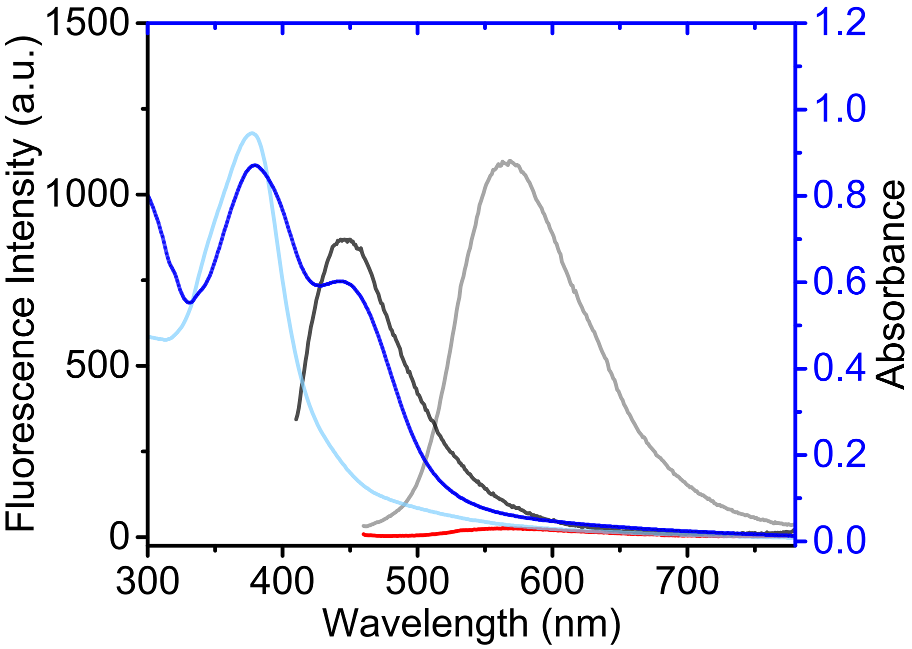

Figure S4. The UV-Vis spectrum of 147 μM **N-I** (light blue line) with the addition of 40 μM $ONOO^-$ (deep blue line), as well as the emission spectrum of 37.2 μg/mL CDs (black line) and 147 μM **N-I** (red line) with 30 μM $ONOO^-$ (grey line). The data was obtained in PBS buffer (5.5 mM, containing 1% DMSO), pH = 7.4 at 25 °C, λ_{ex} = 400 nm (for CDs) or 450 nm (for **N-I**).

Sample Name	Fluorescence Quantum Yield
**CDs**	19.44 %
**N-I**	-0.09%
**N-I** (40 μM $ONOO^-$)	2.04 %
**CD-N-I**	0.93 %
**CD-N-I** (40 μM $ONOO^-$)	2.83 %

Table S1. The fluorescence quantum yields of the CDs we developed (λ_{ex} = 400 nm), **N-I** **(**λ_{ex} = 450 nm), **N-I** with the addition of 40 μM $ONOO^-$ **(**λ_{ex} = 450 nm), **CD-N-I** (λ_{ex} = 400 nm), **CD-N-I** with the addition of 40 μM $ONOO^-$ (λ_{ex} = 400 nm). The data was obtained in PBS buffer (5.5 mM, containing 1% DMSO), pH = 7.4 at 25 °C.

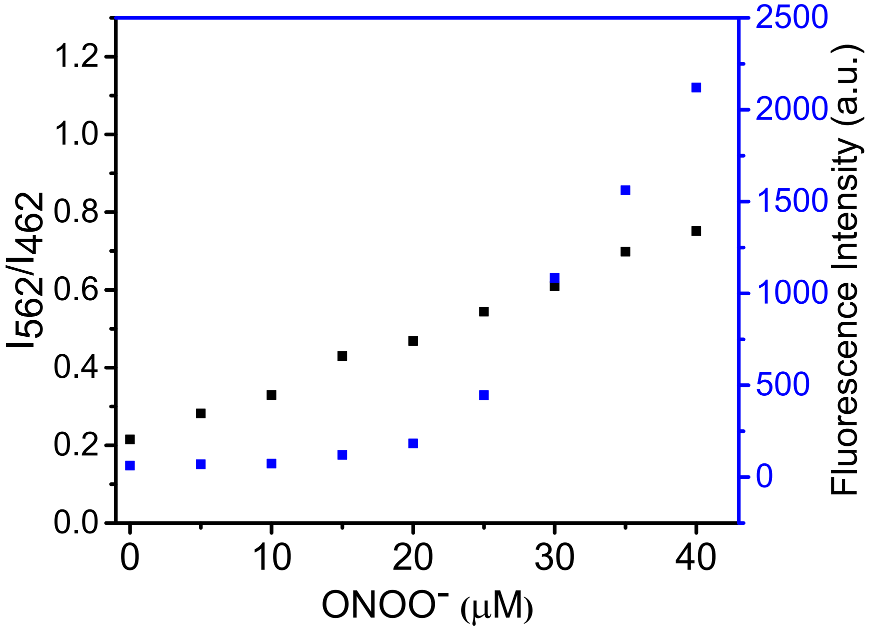

Figure S5. Dose dependence curves for **CD-N-I** (72.3 μg/mL) in black (λ_{ex} = 400 nm, λ_{em} = 462 nm and 562 nm) and **N-I** (147 μM) in blue (λ_{ex} = 450 nm, λ_{em} = 562 nm) in the presence of $ONOO^-$ (0-40 μM). The data was obtained in PBS buffer (5.5mM, containing 1% DMSO), pH = 7.4 at 25 °C.

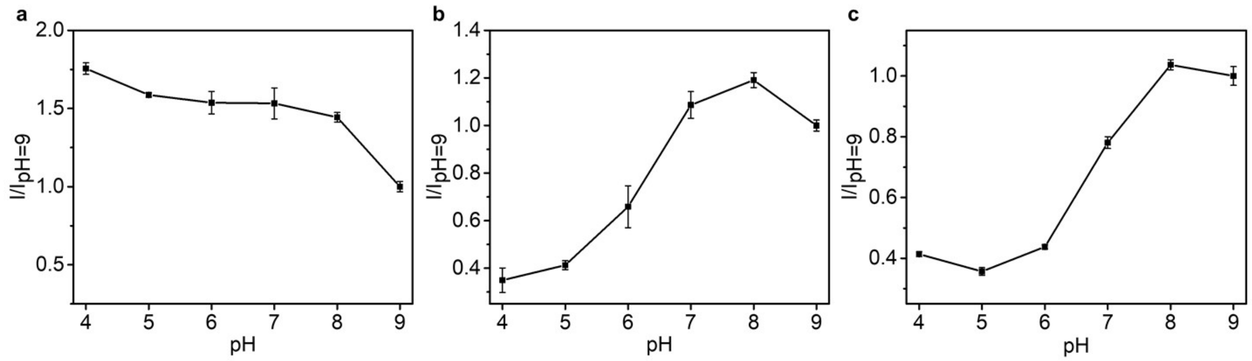

Figure S6. Effects pH left on the fluorescence intensities of 37.2 μg/mL CDs (**a**) and 147 μM **N-I** (**b**) with 40 μM $ONOO^-$ (**c**). The data was obtained in PBS buffer (5.5 mM, containing 1% DMSO), at 25 °C λ_{ex} = 400 nm (for CDs) or 450 nm (for **N-I**), λ_{em} = 462 nm (for CDs) or 562 nm (for **N-I**).

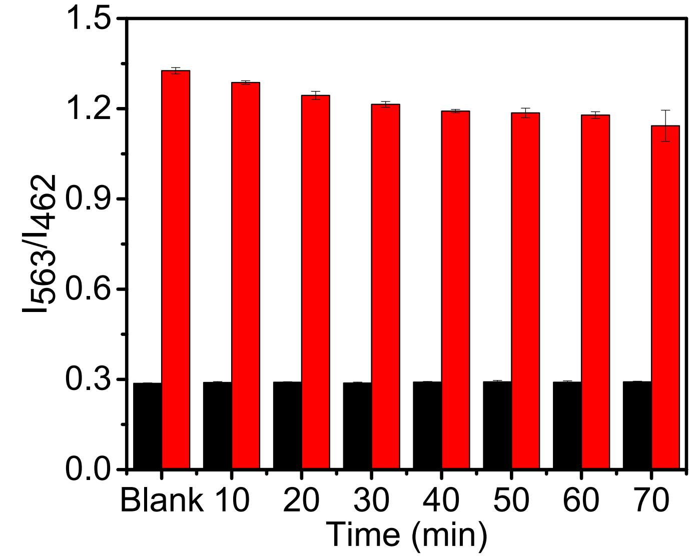

Figure S7. Fluorescence ratio I_{562}/I_{462} for 72.3 μg/mL **CD-N-I** (black bars) in the presence of 40 μM $ONOO^{-}$ (red bars) under 365 nm UV light irradiation, and evaluated after 0 min, 10 min, 20 min, 30 min, 40 min, 50 min, 60 min and 70 min. The data was obtained in PBS buffer (5.5 mM, containing 1% DMSO), at 25 °C λ_{ex} = 400 nm.

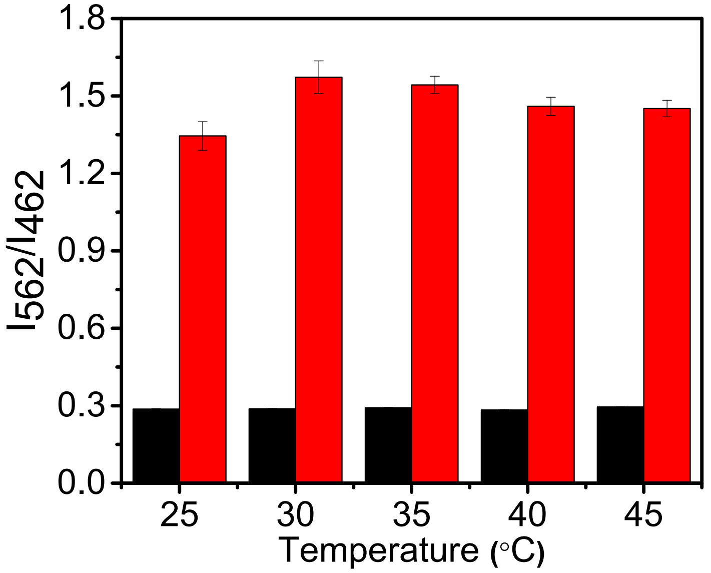

Figure S8. Fluorescence ratio I_{562}/I_{462} for 72.3 μg/mL **CD-N-I** (black bars) in the presence of 40 μM $ONOO^{-}$ (red bars) tested at 25 °C, 30 °C, 35 °C, 40 °C and 45 °C. The data was obtained in PBS buffer (5.5 mM, containing 1% DMSO), λ_{ex} = 400 nm.

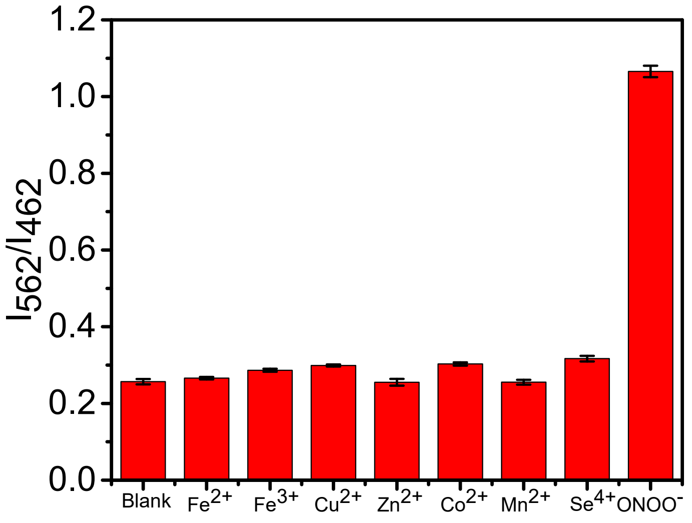

Figure S9. Selectivity data for **CD-N-I** (72.3 μg/mL) in the presence of $ONOO^-$ (40 μM) after 5 minutes, Fe^{2+} (40 μM), Fe^{3+} (40 μM), Cu^{2+} (40 μM), Zn^{2+} (40 μM), Co^{2+} (40 μM), Mn^{2+} (40 μM) and Se^{4+} (40 μM) after 30 minutes. The data was obtained in PBS buffer (5.5 mM, containing 1% DMSO), at 25 °C λ_{ex} = 400 nm.

4. Cell Imaging

4.1. Preparation of **CD-N-I**

CDs were mixed with **N-I** (1.05 mg powder dissolved in 250 μL DMSO) at a ratio of 1:1 and sonicated for 10 min. Then, 50 μL of CD-N-I was added to 1 mL of high glucose DMEM medium which defined as C_1 (72.3 μg/mL).

4.2. Cytotoxicity Assay

The cytotoxicity of **CD-N-I** to HepG2 cells was evaluated by CCK-8. HepG2 cells were cultured using high glucose DMEM medium (10% FBS) in 96 well plates in incubator for 24 h. After that, the medium was exchanged by fresh medium and each well was incubated with a range of **CD-N-I** concentrations (C_1-C_6: 72.3 μg/mL, 36.2 μg/mL, 18.1 μg/mL, 9.0 μg/mL, 4.5 μg/mL, 2.3 μg/mL) for 6 h or 24 h, respectively. Then, CCK- 8 reagent was added and cultured for 1 h, and the absorbance of each well was measured at 450 nm using theEnVision multilabel plate reader (PerkinElmer, USA).

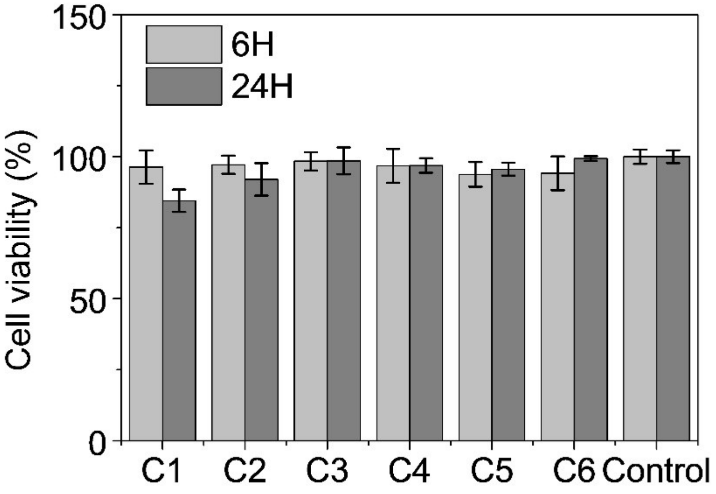

Figure S10. Cytotoxicity of live HepG2 cells incubated with different concentrations of **CD-N-I** (C_1-C_6: 72.3 μg/mL, 36.2 μg/mL, 18.1 μg/mL, 9.0 μg/mL, 4.5 μg/mL, 2.3 μg/mL) for 6 hours and 24 hours.

4.3. Cell Culture and Imaging

HepG2 cells (ATCC® HB-8065™) were maintained in a Dulbecco's Modified Eagle's Medium (DMEM) supplemented with 10 % FBS in a humidified atmosphere of 5 % CO_2 and 95 % air at 37 °C and split when the cells reached 90 % confluency.

HepG2 cells were incubated with **CD-N-I** probe (C_1: 72.3 μg/mL) for 2 h and then incubated with SIN-1 (0, 0.5, 1, 2 mM) for 2 h. To confirm the detection of exogenously generated $ONOO^-$, the cells were pre-incubated with NAC.

To examine whether **CD-N-I** (C_1: 72.3 μg/mL) can be applied to detect endogenous $ONOO^-$ in living cells**,** HepG2 cells were pretreated with 4 mM acetaminophen (APAP) for 24h or 48h, followed by incubating with **CD-N-I** (C_1: 72.3 μg/mL) for 2 h. In the control group, HepG2 cells were treated with APAP and NAC.

Cells were washed with PBS (phosphate buffered saline) three times before imaging. An Olympus FV3000 laser scanning confocal microscope was used to image HepG2 cells with 405 nm excitation. Meanwhile, fluorescence emissions in the blue and green channels were collected in the ranges of 440−500 and 540−600 nm, respectively.

4.4. Imaging exogenous $ONOO^-$

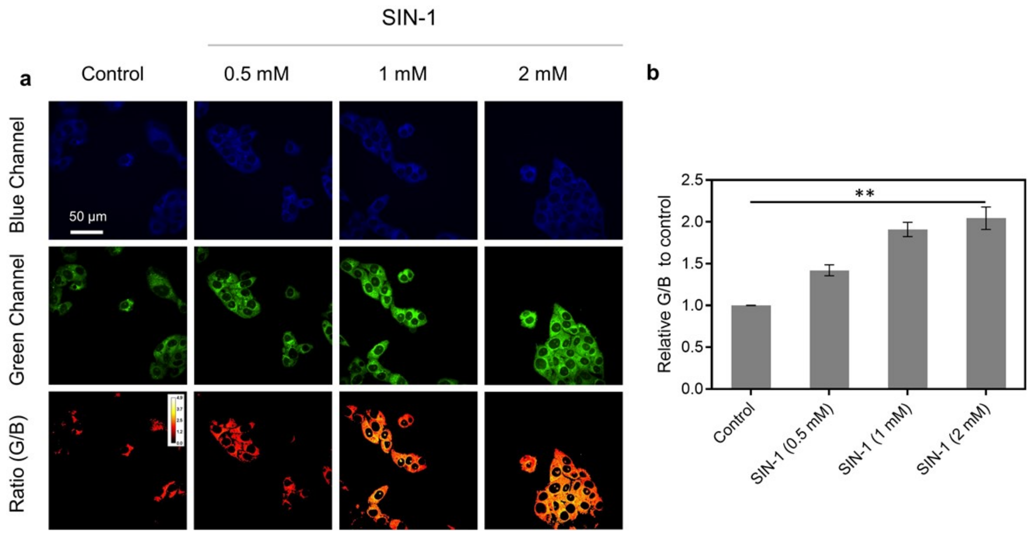

Figure S11. Fluorescence imaging (**a**) and fluorescence quantification (**b**) of HepG2 cells treated with the **CD-N-I** (72.3 μg/mL, 2h) in the presence of SIN-1 (0.5, 1 and 2 mM). Fluorescence data was collected using λ_{ex} = 405 nm, λ_{em} = 440-500 nm and 540-600 nm. ***P* < 0.01. Error bars represent S. D. (n=3).

4.5. Imaging endogenous $ONOO^-$

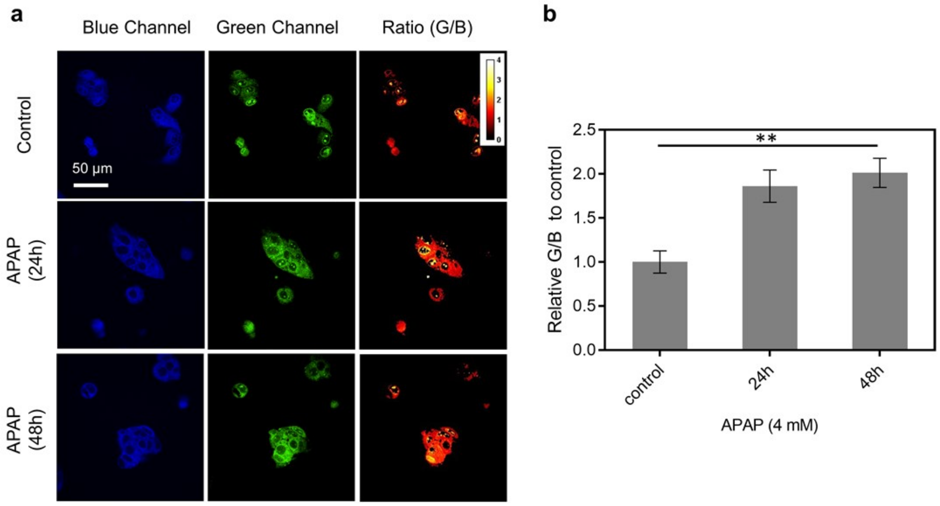

Figure S12. Fluorescence imaging (**a**) and fluorescence quantification (**b**) of HepG2 cells treated with the **CD-N-I** (72.3 μg/mL, 2h) in the presence of APAP (4 mM) for 24h and 48h. Fluorescence data was collected using λ_{ex} = 405 nm, λ_{em} = 440-500 nm and 540-600 nm. ***P* < 0.01. Error bars represent S. D. (n=3).

5. NMR

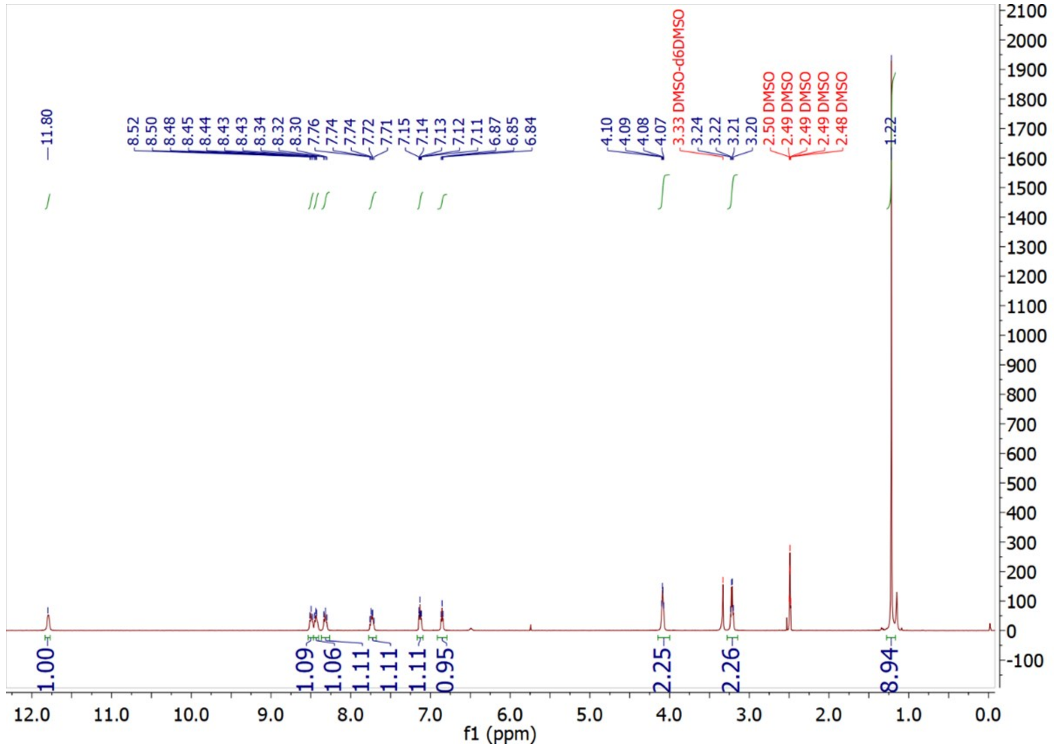

Figure S13. The ^{1}H NMR spectrum of compound **1**.

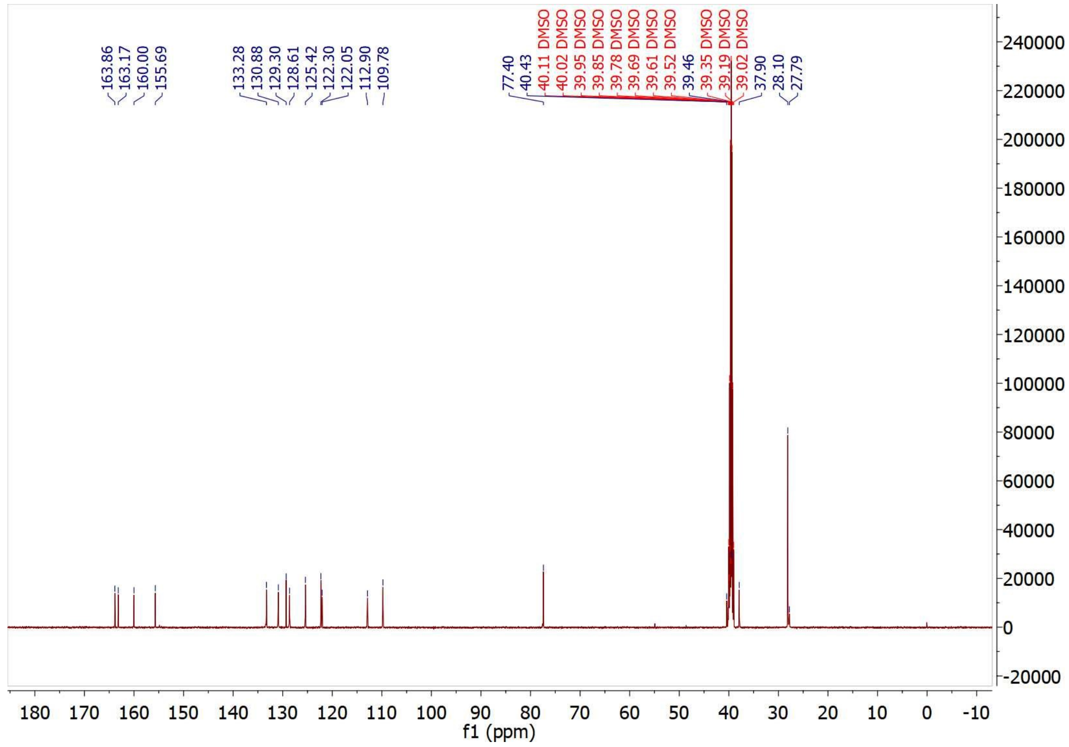

Figure S14. The ^{13}C NMR spectrum of compound **1**.

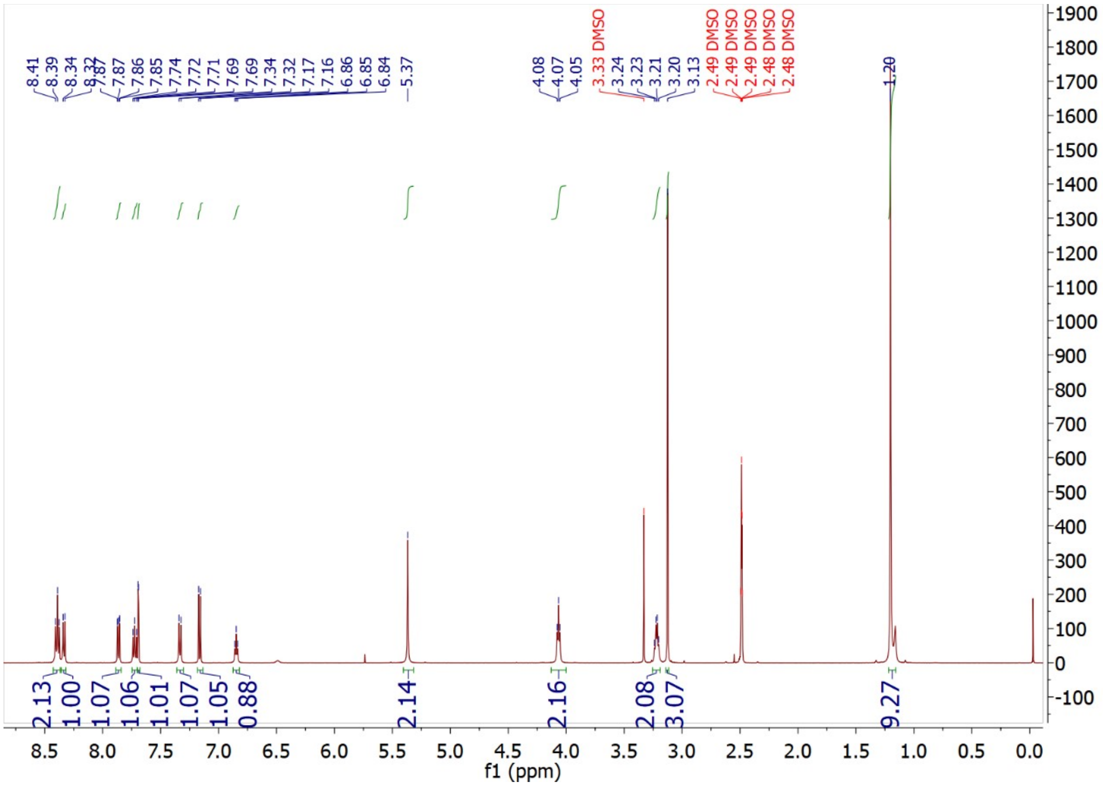

Figure S15. The ^{1}H NMR spectrum of compound **3**.

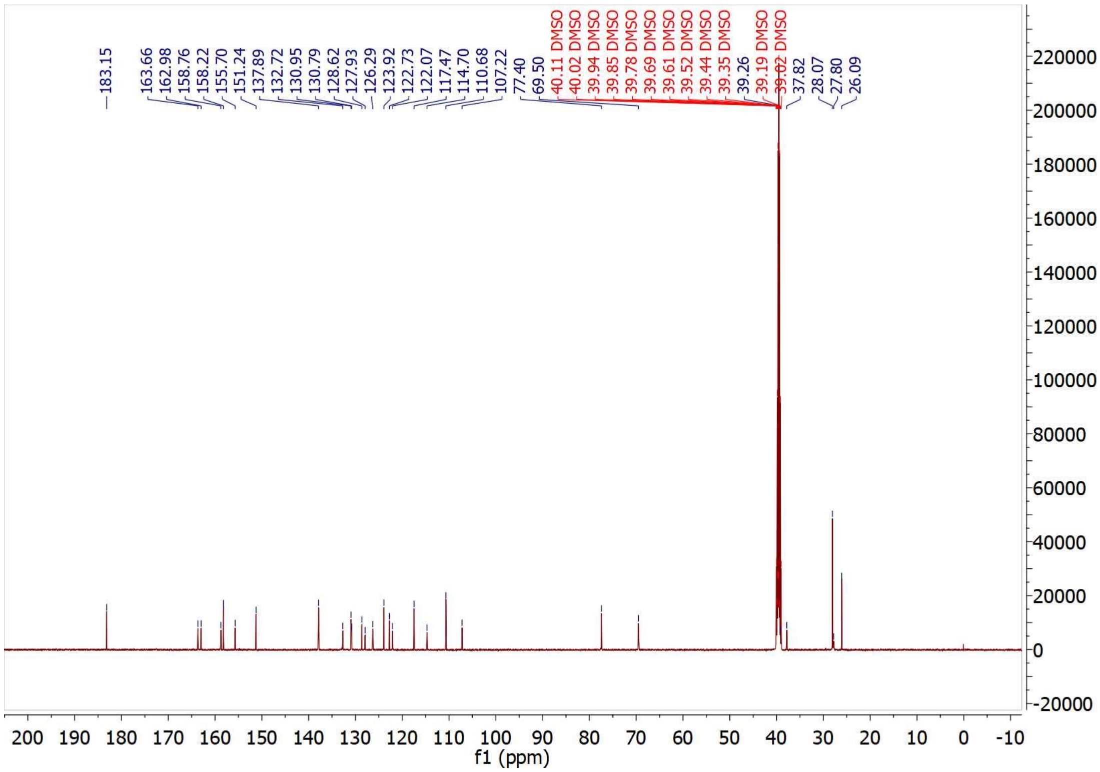

Figure S16. The ^{13}C NMR spectrum of compound **3**.

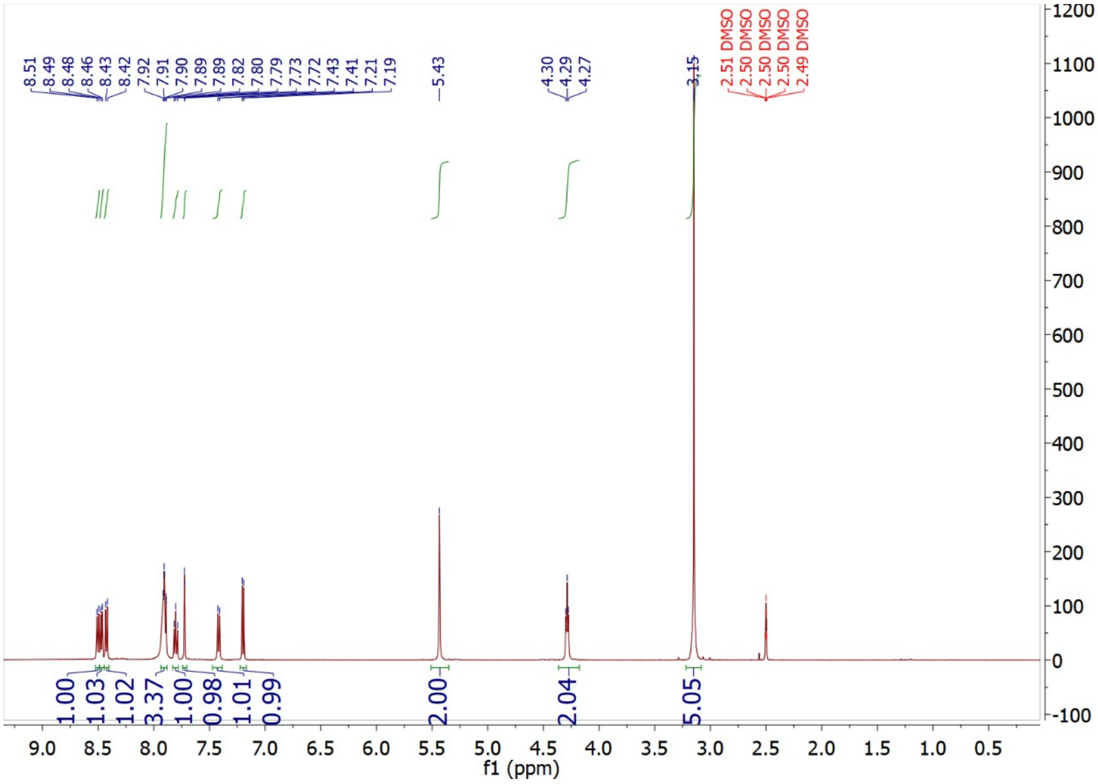

Figure S17. The ^{1}H NMR spectrum of compound **N-I**.

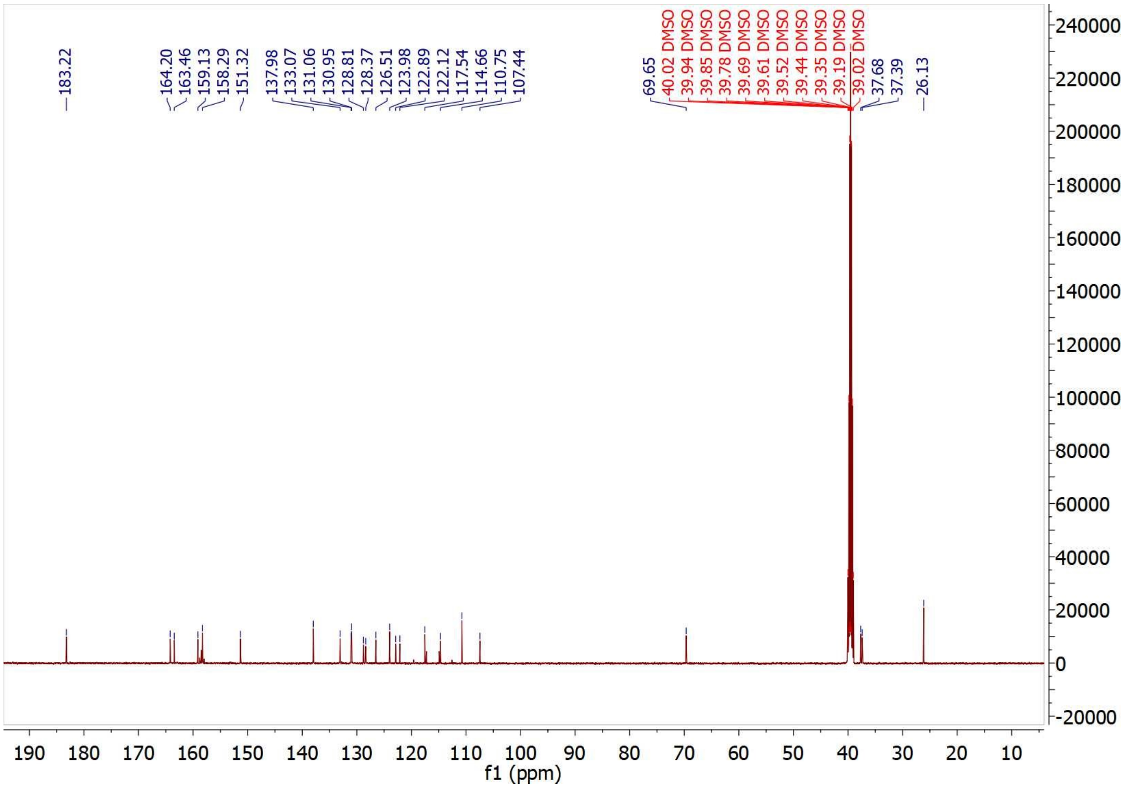

Figure S18. The ^{13}C NMR spectrum of compound **N-I**.

6. References

1. Y. C. Wu, H. H. Han, L. He, L. Li, Y. Zang, J. Li, X. P. He, Y. P. Ding, W. G. Cao and T. D. James, *Chem. Commun.*, 2023, **59**, 5051-5054.
2. J. H. Xiong, W. W. Wang, C. X. Wang, C. Zhong, R. Q. Ruan, Z. Q. Mao and Z. H. Liu, *ACS Sens.*, 2020, **5**, 3237-3245.
3. P. R. Su, Z. W. Zhu, Y. H. Tian, L. J. Liang, W. Y. Wu, J. Cao, B. Cheng, W. S. Liu and Y. Tang, *Talanta*, 2020, **218**, 121127-121134.
4. E. E. Rudebeck, R. P. Cox, T. D. M. Bell, R. Acharya, Z. K. Feng, N. Gueven, T. D. Ashton and F. M. Pfeffer, *Chem. Commun.*, 2020, **56**, 6866-6869.
5. S. Wang, L. Y. Chen, P. Jangili, A. Sharma, W. Li, J. T. Hou, C. Q. Qin, J. Yoon and J. S. Kim, *Coord. Chem. Rev.*, 2018, **374**, 36-54.
6. R. F. Ohana, T. A. Kirkland, C. C. Woodroofe, S. Levin, H. T. Uyeda, P. Otto, R. Hurst, M. B. Robers, K. Zimmerman, L. P. Encell and K. V. Wood, *ACS Chem. Biol.*, 2015, **10**, 2316-2324.
7. X. Zhou, F. Su, H. Lu, P. Senechal-Willis, Y. Tian, R. H. Johnson and D. R. Meldrum, *Biomaterials*, 2012, **33**, 171-180.
8. S. Q. Shen, L. L. Dong, W. Chen, X. D. Zeng, H. Z. Lu, Q. Yang and J. J. Zhang, *ACS Medicinal Chem. Lett.*, 2018, **9**, 1241-1246.
9. J. S. Sidhu, A. Singh, N. Garg and N. Singh, *ACS Appl. Mater. Interfaces*, 2017, **9**, 25847-25856.
10. J. Q. Wang, P. F. Zhang, C. Huang, G. Liu, K. C. F. Leung and Y. X. J. Wang, *Langmuir*, 2015, **31**, 8063-8073.
11. Y. Y. He, Z. X. Li, Q. Y. Jia, B. J. Shi, H. Y. Zhang, L. H. Wei and M. M. Yu, *Chin. Chem. Lett.*, 2017, **28**, 1969-1974.
